# Lack of cerebellar tDCS effects on learning of a complex whole body dynamic balance task in middle-aged (50–65 years) adults

**DOI:** 10.1186/s42466-020-00085-x

**Published:** 2020-09-22

**Authors:** M. Rauscher, F. Yavari, G. Batsikadze, N. Ludolph, W. Ilg, M. A. Nitsche, D. Timmann, K. M. Steiner

**Affiliations:** 1 Department of Neurology, Essen University Hospital, University of Duisburg-Essen, Hufelandstr. 55, 45147 Essen, Germany; 2 Department of Psychology and Neurosciences, Leibniz Research Centre for Working Environment and Human Factors, Dortmund, Germany; 3 Cognitive Neurology, Section Computational Sensomotorics, Hertie Institute for Clinical Brain Research and Center for Integrative Neuroscience, Eberhard Karls University, Tübingen, Germany; 4 Department of Neurology, University Medical Hospital Bergmannsheil, Bochum, Germany

**Keywords:** Cerebellum, Transcranial direct current stimulation, Motor learning, Aging

## Abstract

**Background:**

Cerebellar transcranial direct current stimulation (tDCS) is widely considered as a promising non-invasive tool to foster motor performance and learning in health and disease. The results of previous studies, however, are inconsistent. Our group failed to provide evidence for an effect of cerebellar tDCS on learning of a complex whole body dynamic balance task in young and healthy participants. Ceiling effects in the young study population are one possible explanation for the negative findings.

**Methods:**

In the present study, we therefore tested 40 middle-aged healthy participants between the ages of 50 to 65 years. Participants received either anodal or sham cerebellar tDCS using a double-blinded study design while performing a balance task on a Lafayette Instrument 16,030 stability platform®. Mean platform angle and mean balance time were assessed as outcome measures.

**Results:**

Significant learning effects were found in all participants. Balancing performance and learning rate was significantly less in the group of middle-aged adults compared to our previous group of young adults. No significant effects of cerebellar tDCS were observed.

**Conclusions:**

Our findings are in line with other studies that have failed to prove robust effects of cerebellar tDCS on motor learning. The present findings, however, do not exclude cerebellar tDCS effects. tDCS effects may be more prominent after repeated stimulation, using other stimulus parameters, in patient populations, or in other motor learning tasks.

**Trial registration:**

Not applicable.

## Background

The cerebellum is essential for motor performance and motor learning [44]. Motor functions decline not only in cerebellar disease but also in physiological aging. An age-related reduction of cerebellar volume is well known and starts around the age of 40 years [29]. Balance and gait deteriorate with age [22, 23]. Likewise, an age-related decline of motor learning is well known [1, 5] and age-related reduction of cerebellar function is one likely factor. For example, older adults exhibited lower activation in cerebellar lobule VI than young adults in a motor sequence learning task [5]. Transcranial direct current stimulation (tDCS) has received increasing attention over the last two decades as a non-invasive technique to modulate neuroplasticity. More recently, the cerebellum has been identified as a possible target of tDCS [28, 33]. Several studies have shown that cerebellar tDCS modulates cerebellar brain inhibition (CBI) [2, 13] a physiological measure which quantifies the inhibitory projection from the cerebellar cortex to the motor cortex via the dentato-thalamo-cortical pathway [42]. Cerebellar tDCS has also been shown to modify fMRI activity of the cerebellar cortex [24] and cerebellar nuclei [20]. With respect to age-related deterioration and furthermore, in cerebellar disease, tDCS may have beneficial effects [3, 4]. For example, adaptive postural control has been shown to be improved by anodal cerebellar tDCS in healthy volunteers [27]. Anodal cerebellar tDCS has been shown to improve locomotor adaptation in a split-belt paradigm (2011). Furthermore, Cantarero et al. [7] demonstrated faster acquisition of a sequential visual isometric pinch task with anodal cerebellar tDCS. Hardwick and Celnik [16] reported that cerebellar tDCS was able to compensate for age-related decline in a reach adaptation task. Cerebellar tDCS, however, did not improve acquisition of a complex whole body dynamic balance task [36]. Young and healthy participants were tested who possibly performed already at maximum possible learning rate preventing the observation of tDCS effects. Therefore, we re-examined possible cerebellar tDCS effects in the same dynamic balance task in middle-aged and healthy adults.

## Methods

### Participants

Forty healthy participants (20 male, 20 female) aged 50–65 years took part in this study. Participants were pseudorandomly assigned to two groups – a verum stimulation group and a sham stimulation group. Male and female participants were equally distributed across groups. None of the participants suffered from any neurological, psychiatric or orthopedic disorders or were taking centrally acting medication. All participants were non-smokers, except one. All participants were examined by an experienced neurologist (MR) on the day of the experiment. Clinical assessment included the International Cooperative Ataxia Rating Scale [ICARS [41]] and the Scale for the Assessment and Rating of Ataxia [SARA [34]]. The neurological examination was unremarkable in all participants. In a previous study of our group [37], male participants performed worse than female correlating with body height. Because foot position was fixed, biomechanical constraints likely explain the lower performance in taller participants. Therefore, participants taller than 190 cm were excluded from the present study. The study was approved by the local ethics committee (Medical Faculty of the University Duisburg-Essen) and conducted in accordance with the Declaration of Helsinki.

### Dynamic balance task

**Fig. 1:**
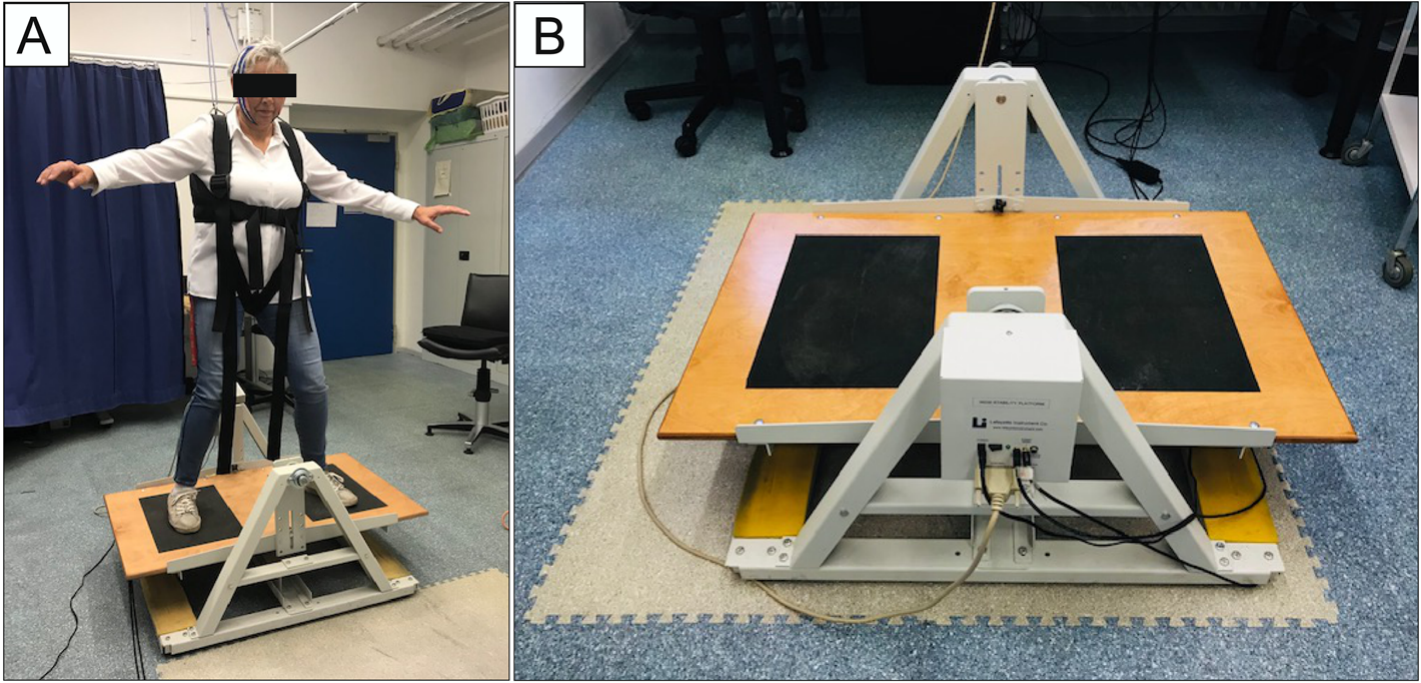
Experimental set-up. **a** Middle-aged woman (57 years old) performing the complex whole body dynamic balance task. Informed written consent has been given for publishing the picture. **b** Lafayette Instrument 1600 stability platform®

The dynamic balance task is described in Steiner et al. [36] in more detail. The Lafayette Instrument 16,030 Stability Platform® was used for balance training (Fig. 1) [19]. Participants stood on the platform and were instructed to hold it in a horizontal position as long as possible. The participants were strapped into a safety harness during training. They performed 20 trials lasting 30 s each. The platform was lowered to the ground towards alternating sides for 30 s between the trials in order to avoid muscle fatigue. The mean platform angle deviation was measured during each trial by an analog-to-digital converter (National Instruments, Germany) at 1 kHz. Furthermore, the mean balance time, which is defined as the time during which the platform could be held between − 5° and + 5°, was calculated.

### Cerebellar transcranial direct current stimulation

Cerebellar tDCS was applied by a neuroConn DC-stimulator at a current intensity of 2.8 mA to allow for the same current density (0.08 mA/cm^2^) as in Celnik et al. [[16], see also [13, 14]]. An electrode of the size of 5 cm × 7 cm was placed above the cerebellum. The electrode was horizontally oriented. The upper edge of the electrode was located 0.5 cm above the inion (center 2 cm below the inion) [11]. Two return electrodes (5 cm × 5 cm) were placed over each of the buccinator muscles [36]. The electrode positions were chosen with the aim of targeting both the vermis and the cerebellar hemispheres [36]. Ten20 conductive paste was used to affix the electrodes. In addition, cotton straps were wrapped around the head and the electrodes. tDCS was applied during training on the platform. The stimulator was switched on at the time the participant started the first trial (after the test trial) and stimulation lasted until the last trial. Thus, the duration of the stimulation was 19 min and thirty seconds (20 trials à 30 s and 19 breaks à 30 s between trials). tDCS was applied in a double-blinded fashion, i.e. neither the examiner nor the participant knew whether verum or sham stimulation was applied. One group received anodal tDCS (stimulation group) whereas the other group received sham stimulation. In sham stimulation, the current was ramped up in 10 s and remained at 2.8 mA for a duration of 30 s. tDCS started after the first trial. The first trial served as test trial to control for possible differences in performance between the two groups.

Modeling of tDCS electric field (EF) distribution was performed using SimNIBS, a freely available software package for simulating the effects of non-invasive brain stimulation (NIBS) techniques [40]. A realistic head model was created using T1- and T2-weighted average MRI templates in the age range of 55–59 years taken from the Neurodevelopmental MRI Database [[30] https://www.nitrc.org/projects/neurodevdata/]. Three electrodes covered with conductive gel were positioned over the same areas as in the experimental procedure (one 5 cm × 7 cm electrode centered 2 cm below the inion, and two 5 cm × 5 cm electrodes over the buccinator muscles). The current intensity was set to 2.8 mA. The calculated normEF values were converted to nii using the msh2nii command and exported to Matlab (R2019a, version 9.6.0, The MathWorks Inc.). Then, using cerebellar masks extracted from the SUIT atlas [9] the average value of normEF strength was quantified in four cerebellar regions that are assumed to be involved in the task (i.e., i) vermis and anterior lobe, ii) Crus I, iii) Crus II and iv) lobule VIII, bilaterally).

### Statistical analysis

An analysis of variance (ANOVA) with repeated measures was applied in order to test for group differences, with mean platform angle deviation and mean balance time as dependent variables, trials (1–20) as within subject factor and stimulation (verum vs. sham) as between subject factor. Results were considered significant at *p* < 0.05. Degrees of freedom were adjusted, if applicable, in accordance with Greenhouse and Geisser [15]. Statistical tests were performed using SPSS software (version 17, IBM Company, New York, USA). The Two One-Sided Test (TOST) procedure was applied to test for equivalence between the anodal and sham group [31, 35]. Equivalence was assumed when the 90% confidence interval for the differences between the means of the anodal and sham groups fell within the range of +/− 1 standard deviation of the respective mean of the sham group.

## Results

### Modeling of tDCS electric field (EF) distribution

**Fig. 2:**
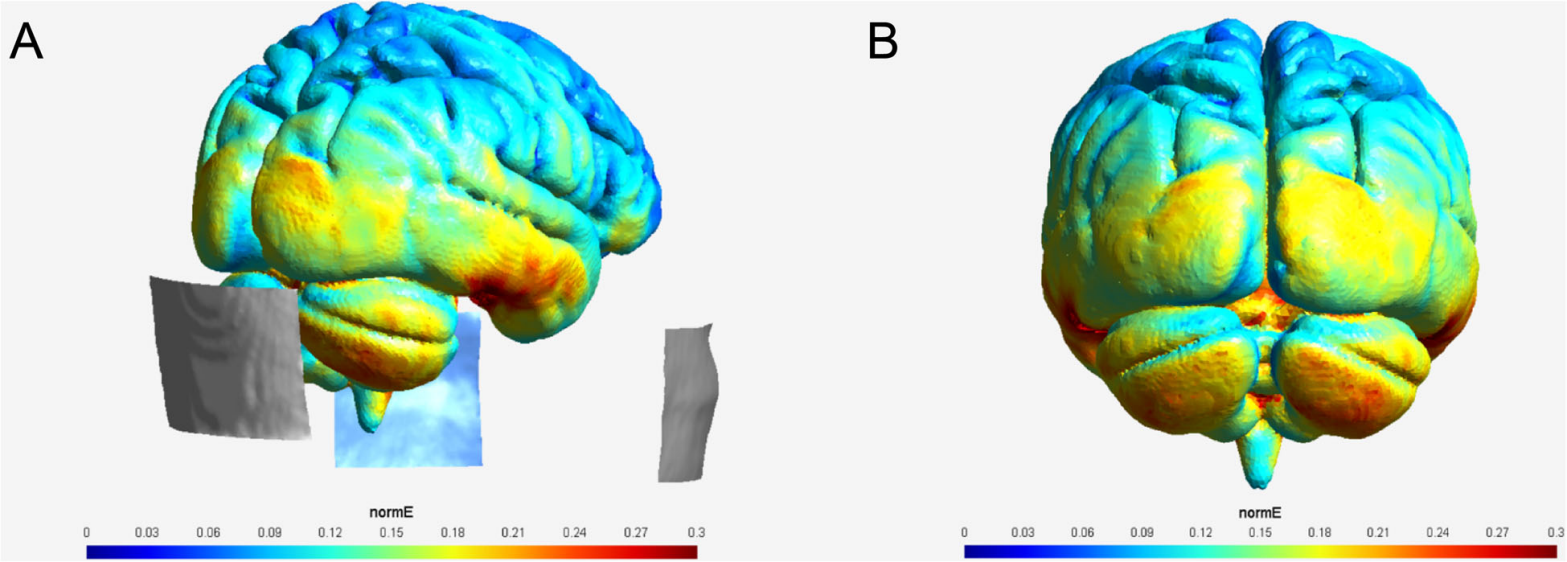
Modelling results of cerebellar tDCS effects. Distribution of normEF values (V/m) calculated using SimNIBS overlayed on an average brain in the age range of 55–50 years [taken from Neurodevelopmental MRI Database [30]; https://www.nitrc.org/projects/neurodevdata/]. **a** Lateral view, showing also the target electrode over the cerebellum and return electrodes over the buccinator muscles. **b** View from the back

Cerebellar stimulation effects as revealed by modelling of the electric field showed the highest values for Crus II bilaterally [0.78 V per meter (V/m)] and lobule VIII bilaterally (0.34 V/m), and lower values for Crus I bilaterally (0.23 V/m) and the vermis/anterior lobe (0.19 V/m) (Table 1). Distribution of normEF values is shown in Fig. 2 overlayed on an average brain [age range 55–50 years, taken from the Neurodevelopmental MRI Database [30]; https://www.nitrc.org/projects/neurodevdata/]**.**

**Table 1:** Mean values of the electric field (normEF) in four different regions of the cerebellum based on the SUIT atlas [9] calculated using SimNIBSc [32]

	Mean of normEF (V/m)	Labels of the included cerebellar regions from SUIT atlas
**Vermis plus the anterior lobe of cerebellum**	0.1782040	1–4, 6, 9, 12, 15, 18, 21, 24, 27
**Crus I - both hemispheres**	0.2315656	10, 8
**Crus II - both hemispheres**	0.7774634	13, 11
**Lobule VIII - both hemispheres**	0.3386466	17, 19, 20, 22

### Dynamic balance task

**Fig. 3:**
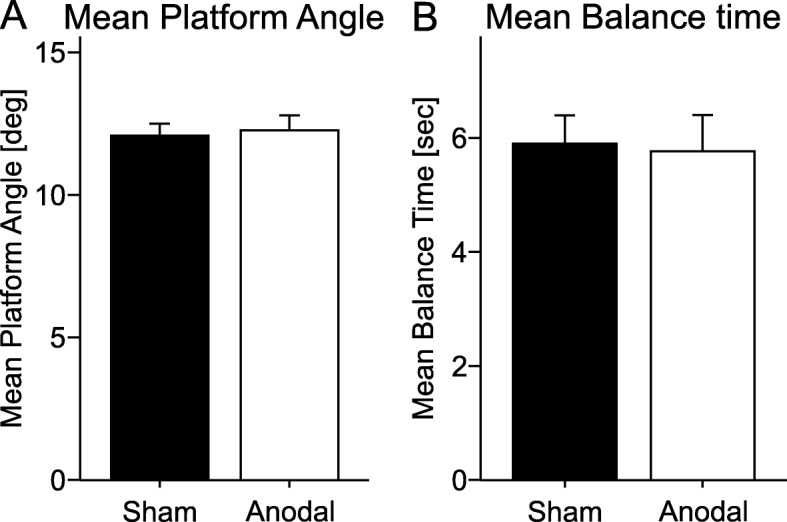
Test trial with no stimulation applied. Mean platform angle and standard error of the mean, and mean balance time and standard error of the mean in the test trial in each stimulation group (verum vs. sham)

There was no significant difference regarding age or body height between the two stimulation groups [unpaired t-test: age: anodal group: mean 58.45 ± 4.7 years, sham group: mean 56 ± 4.35 years; t (38) = 1.71, *p* = 0.63; body height: anodal group: mean 170.25 ± 7.06 cm, sham group: mean 169.3 ± 7.43 cm; t (38) = 0.42, *p* = 0.93]. There was no significant group difference in performance of the test trial without stimulation [mean platform angle: t (38) = 0.05, *p* = 0.42; mean balance time: t (38) = − 0.66, *p* = 0.37; Fig. 3].

**Fig. 4:**
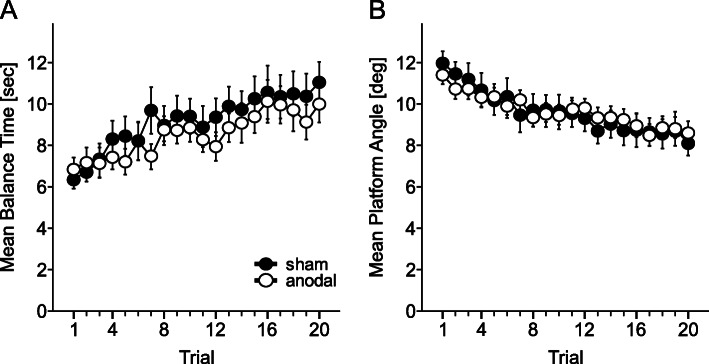
Mean balance time and standard error of the mean (**a**), and mean platform angle and standard error of the mean (**b**) across the 20 learning trials. Black: sham group, white: anodal group

All participants improved over the course of the experiment (Fig. 4). Mean platform angle significantly decreased and mean balance time significantly increased across trials [trial effect: mean platform angle: F (19,722) = 27.4, *p* < 0.001; mean balance time: F (19,722) = 10.23, p < 0.001]. There was no significant difference between the stimulation groups [stimulation effect: mean platform angle: F (1,38) = 0.002, *p* = 0.96; mean balance time: F (1,38) = 0.44, *p* = 0.51] and no significant trial by stimulation interaction [mean platform angle: F (19,722) = 1.53, *p* = 0.68, mean balance time: F (19,722) = 0.82, *p* = 0.69]. Including body height as a covariate in the ANOVA with repeated measures revealed similar results (group, trial, group by trial, height effects, all *p* values > 0.1).

The mean differences between the means over all trials in the anodal and the sham group were found to be equivalent [t (32,48) = 3.71, *p* = 0.0004; equivalence bounds: ± 3.12, alpha: 0.05].

**Fig. 5:**
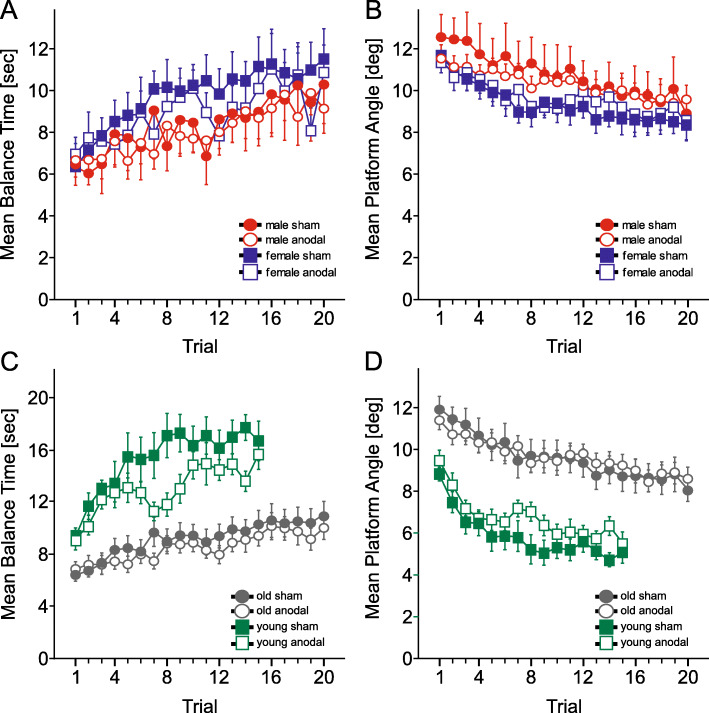
Mean balance time and standard error of the mean (**a**, **c**) and mean platform angle and standard error of the mean (**b**, **d**) in each stimulation group, considering male and female participants separately (**a**, **b**) and comparing old and young participants in each stimulation group (**c**, **d**). Red circles: Male participants sham, white circles: male participants anodal, blue squares: female participants sham, white squares: female participants anodal. Grey circles: old participants sham, white circles: old participants anodal, green squares: young participants sham, white squares: young participants anodal

Numerically, male participants performed somewhat lower than female participants (Fig. 4). Therefore male and female participants were analyzed separately. Again, there were no significant differences between the anodal and sham groups [male participants only: mean platform angle: stimulation effect: F (1,18) = 0.12, *p* = 0.73; mean balance time: stimulation effect: F (1,18) = 0.04, *p* = 0.84; female participants only: mean platform angle: stimulation effect: F (1,18) = 0.13, p = 0.73; mean balance time: stimulation effect: F (1,18) = 0.34, *p* = 0.57]. Trial by stimulation interactions were not significant, with trendwise effects regarding the mean platform angle in male participants [male participants only: mean platform angle: trial by stimulation interaction: F (19, 342) = 1.61, *p* = 0.051; mean balance time: trial by stimulation interaction: F (19,342) = 0.73, *p* = 0.79; female participants only: mean platform angle: trial by stimulation interaction: F (19,342) = 0.84, *p* = 0.66; mean balance time: trial by stimulation interaction: F (19,342) = 0.82, *p* = 0.68] (Fig. 5a, b). Trial effects were significant (all p values < 0.001). Male participants were significantly taller than female participants [male participants: mean: 175.1 ± 5.2 cm, female participants: mean: 164.5 ± 4.5 cm; t (38) = 6.89, *p* < 0.001]. There was however no significant correlation between body height and task performance (mean platform angle, mean of all trials, Pearson’s correlation with body height: r = 0.09, *p* = 0.58; mean balance time, mean of all trials, Pearson’s correlation with body height: r = − 0.12, *p* = 0.48).

Finally, we compared the group of all participants included in the present study to with the young healthy participants of our previous experiment [36]. Note that the experimental set-up, training paradigm and stimulation protocol was identical in the present study and the previous study, with the only exception of trial number, which was 15 trials in the previous study compared to 20 trials in the present study. The older adults performed significantly worse than the young healthy adults [mean platform angle (mean of the first 15 trials): young adults: 6.43 ± 1.57 degrees, older adults: 10.1 ± 2.61 degrees; group effect: F (1,68) = 45.5, *p* < 0.001; mean balance time (mean of the first 15 trials): young adults: 14.03 ± 3.44 s, older adults: 8.41 ± 3.04 s, group effect: F (1,68) = 52.4, *p* < 0.001] (Fig. 5c, d). Learning rate was significantly higher in the young participants [trial by age interaction: mean platform angle: F (14, 660) = 1.96, *p* = 0.035; mean balance time: F (14, 663.06) = 3.77, *p* < 0.001]. The trial effect was significant [trial effect: mean platform angle: F (14,660) = 47.77, *p* < 0.001; mean balance time: F (14,663) = 25.7, p < 0.001]. In young participants (squares in Fig. 5 c, d), the anodal group performed numerically below the sham group, but this difference was not significant [mean platform angle: F (1,18) = 2.4, *p* = 0.12; mean balance time: F (1,18) = 3.72, *p* = 0.07].

## Discussion

In the present study, anodal cerebellar tDCS had no effect on learning of a complex whole body dynamic balance task in healthy middle-aged adults. All participants were able to improve balancing over the twenty trials, measured by a significant increase in the mean balance time and a significant decrease in the mean platform angle, but without a significant difference between the stimulation and the sham group. The elderly participants performed significantly worse compared to the young healthy participants in our first study [36]. Thus, as expected, aging affected balancing capability and learning in this task. However, similar to our previous negative findings in young healthy participants, there was no significant effect of cerebellar tDCS in middle-aged adults. In young participants there was a trend that anodal stimulation impeded learning.

The present findings are in line with other studies on cerebellar tDCS showing no or inconsistent effects, not only with regard to balance function but also in other types of motor learning: Jalali et al. [18] conducted seven reach adaptation experiments varying task parameters in a large population of healthy young participants. No consistent cerebellar tDCS effects were obtained. Positive effects in one experiment could not be replicated in a second experiment using the same experimental set-up, paradigm and stimulation parameters. Likewise, two studies of our group [17, 25] found no effect of cerebellar tDCS in a visuomotor reach adaptation task. Kaminski et al. [19] investigated the effect of tDCS in the same whole body dynamic balance task as in the present study but with different electrode positioning: the supplementary motor area was chosen as the stimulation target, as it is an area assumed to control multi-joint whole body movements. Again, tDCS had no beneficial effect on balance learning [19].

Several reasons for the inconsistency of cerebellar tDCS effects have to be considered. Stimulation parameters such as current intensity, electrode placement and electrode size [28] might have been suboptimal with respect to either the stimulation target and/or task. One may argue that the tDCS montage did not sufficiently stimulate the cerebellar areas involved in the task. In fact, modelling of the electric field distribution suggests that cerebellar tDCS effects on the vermis and anterior cerebellar lobe were small. Cerebellar tDCS effects, however, were strong in Crus II bilaterally, but are also present in lobules VIII. Lobule VIII has been shown to be involved in the task by Taubert et al. [39]. The authors examined learning-related grey and white matter changes using the same dynamic balance task. Grey matter (GM) volume in the left lobule VIII correlated negatively with improvements in motor performance. Likewise, a GM decrease was found in lobule VIII bilaterally, and a mean diffusivity (MD) increase in right cerebellar white matter regions. Taubert and colleagues discussed “synaptic pruning, decreased synapse head-size due to long-term depression or proliferation of intracortical axons as possible mechanisms underlying grey matter reduction” [supplementary materials in [39]]. Because strategic learning plays a significant role in successfully performing the task [6, 39], we believe that Crus II likely plays a role because of its known connections with the prefrontal cortex [10, 38]. Thus, although negative findings may be explained because tDCS effects on the vermis were minor, parts of the cerebellum, which are involved in the task, have been stimulated.

Furthermore tDCS effects may be more prominent in multiple session compared to single stimulation session studies [3]. There is also an ongoing discussion about individual differences in responsiveness to tDCS [21]. In addition, and may be most importantly, the highly convoluted structure of the cerebellar cortex makes it difficult to predict the overall tDCS effect which depends on current flow direction relative to the orientation of the axons [8], and may result in antagonistic effects dependent on the depth of penetration of the current [2]. Genetic polymorphisms might be another explanation for the conflicting results in cerebellar tDCS studies. The BDNF Val66Met polymorphism is thought to be associated with slowed motor skill learning due to altered long-term potentiation conditions [12, 26]. Van der Vliet and colleagues found susceptibility of non-carriers to anodal tDCS in eyeblink conditioning [43]. Finally, effect sizes of cerebellar tDCS may be much smaller than initially thought, and consistent tDCS effects may only be picked up in very large study populations [18].

In the present study, the performance of male participants appeared to be worse than the performance of female participants (Fig. 5). Similar to our previous study in young participants, this numerical difference was not significant [36]. We assume that significant differences in body height explain this gender difference. A significant correlation with height was found in a previous pilot study including participants with a height above 190 cm [37] (which were excluded in the present study).

## Conclusions

In conclusion, we observed a lack of cerebellar tDCS effects on learning of a complex whole body dynamic balance task in middle-aged adults. Findings confirmed our previous negative findings in young healthy adults. However, further studies are needed before firm conclusions about cerebellar tDCS effects on motor learning tasks can be drawn.

## Data Availability

The datasets used and/or analysed during the current study are available from the corresponding author on reasonable request.

## Abbreviations

ANOVA: Analysis of variance
EF: Electric field
Fig: Figure
GM: Grey matter
ICARS: International Cooperative Ataxia Rating Scale
MD: Mean diffusivity
MRI: Magnetic Resonance Imaging
SARA: Scale for the Assessment and Rating of Ataxia
tDCS: Transcranial direct current stimulation
TOST: The Two One-Sided Test
